# Routine Enquiry for Domestic Violence during Antenatal Care: An Opportunity to Improve Women's Health

**DOI:** 10.1055/s-0042-1742735

**Published:** 2022-05-16

**Authors:** Fernanda Garanhani Surita, Odette del Risco Sánchez

**Affiliations:** 1 Department of Obstetrics and Gynecology, Universidade Estadual de Campinas, Campinas, SP, Brazil

Violence against women has been recognized by the World Health Organization (WHO) as a health problem and a human rights violation with epidemic proportions which requires an urgent action. 1 2 Worldwide it is estimated that ∼1 in 3 women have suffered physical and/or sexual violence by an intimate partner or non-partner sexual violence in their lifetime. 3 The United Nations considered as violence against women “any act of gender-based violence that results in, or is likely to result in, physical, sexual, or mental harm or suffering to women, including threats of such acts, coercion or arbitrary deprivation of liberty, whether occurring in public or in private life.” 4

Domestic violence (DV), family violence (FV) and intimate partner violence (IPV) are terms frequently observed in the literature. 5 Unfortunately, domestic environment it is a place where many women might suffer violence perpetrated by relatives, former or current partner, showing that particularly their homes could be unsafe places from many women around the world.

In Brazil, recent statistics from Brazilian Forum of Public Security show that 230 160 women disclosed domestic violence and 1.350 feminicides occurred during 2020. 6 If we observed victims’ sociodemographic characteristics it is appreciated a higher number of young women of reproductive health age which are particularly vulnerable to experience diverse forms of violence.

Throughout pregnancy violence episodes could be more frequent and DV might increase during the pregnancy course as well as in the postpartum period. 7 On the other hand, other authors have shown that women with history of violence reported an apparently decrease of DV episodes during pregnancy. 3 8 This variability suggest that some changes in severity and frequency of violence may occur during this period and pregnant woman could be experiencing forms less explicit of violence. However, identify those pregnant women might be suffered current or past experiences of DV contribute to understand the importance to be awareness of this issue and the necessity to provide an appropriate approach during routine antenatal care (ANC). Accordingly, it has been recognized that DV might be more prevalent during pregnancy compared with other conditions such preeclampsia and gestational diabetes commonly screening during this period. 9

There is a consensus regarding history of violence as a risk factor to experiences futures episodes and several evidence confirm that many forms of violence might be experienced during pregnancy and postpartum period. 7 8 10 11 12 Also, when observed these patterns, it is necessary highlight the importance to observe domestic violence as a continuum. 7 In this sense, it is important to recognize that violence can be perform as a cycle and episodes of violence could be recurrent through women’s lifetime.

Regarding the prevalence of physical violence during pregnancy a WHO Multi-Country Study on Women's Health and Domestic Violence against Women observed a variability among countries 1% in Japan city to 28% in Peru province. 3 A Brazilian study conducted among pregnant undergoing ANC in basic health care units of the Brazilian Health System (SUS), revealed that 19.1% ( *n* = 263) reported psychological violence and 6.5% ( *n* = 89) disclosed physical/sexual violence. 11 However, similarly to global estimates, the prevalence of DV among pregnant varied among studies conducted in Brazil.

Furthermore, a widely body of evidence has been shown serious consequences of DV to women wellbeing, particularly to sexual and reproductive health such as neonatal low-birth weight, unintended pregnancies, sexually transmitted infections, and preterm birth. 13 Also, mental health problems such as depression, anxiety, post-traumatic stress disorder and adverse consequences in mother-child bond 14 are described as some consequences of DV experiences during pregnancy.

DV it is a complex phenomenon, and it is needed develop profounder analysis about their dynamics. In this sense, we highlight the importance to improve understanding about DV dynamic based on ecological perspective recognizing that individual, relationship, community and society factors are necessary to achieve a comprehensive approach and consequently implementing strategies to response and prevent violence in all their forms. 3 15

Underreporting DV is common and most of survival have fears, feel worried about safety and face barriers and difficulties to disclose DV experiences. 1 16 However, health system has been recognized as a key sector and those women who experienced DV might show clinical conditions associated with past and/or current DV experiences. Health services are often the first contact for survivors of violence and some clinical conditions associated with IPV experiences could be identified during obstetrics consultations such as unexplained reproductive symptoms, including pelvic pain, sexual dysfunction, repeated vaginal bleeding and sexually transmitted infections, unexplained genitourinary symptoms and chronic pain among others. 17

Health sector response including strategies focused on primary, secondary and tertiary prevention undoubtedly requires a multidisciplinary approach. In the last years WHO have been developed strategies to strengthen the role of the health system including a multisectoral response to address interpersonal violence, especially against women and girls. 2 Women survivors of violence may have a safety, financial, psychosocial, legal protection and health needs 1 and for this reason it is important to recognize the role to addressing DV through implementing public policies, legal services, preventive health care services and psychosocial support.

Several international organizations, including the World Health Organization and International Federation of Gynecology and Obstetrics (FIGO), have developed statements, ethical guidance and recommendations regarding DV and IPV in healthcare settings. The 2013 WHO guideline included a series of minimum conditions to address violence in this context such as a protocol, training for providers, private setting, confidentiality and a system for referral. 17 In addition, particularly during pregnancy has been recognized that IPV it is a relevant issue to be address considering that implementing an appropriate enquiry during antenatal care contribute to a positive experience during this period. 18 In addition, professional organizations such as American Medical Association (AMA), American Academy of Family Physicians (AAFP), Emergency Nurses Association (ENA) and American Academy of Pediatricians (AAP) among others recognized the importance to screening for violence experiences. For instance, American College of Obstetricians and Gynecologists (ACOG) agreed that during pregnancy it is necessary to implementing a screening systematically. In this sense, they recommend routine enquiry during prenatal care and extended to postpartum period. 19 Similarly, FIGO has issued a publication entitled *Ethical guidance on healthcare professionals' responses to violence against women* reinforcing the consequences of DV experiences and the role of gynecologists and obstetricians to address this issue. 20

Gynecologists and obstetricians should play an important role in identification, provide quality information, support, and referral survivals of DV. In this sense, routine enquiry during ANC has been recognized as an opportunity to offer supporting and quality information, helping access resources and validating women’s history. 17 20 The systematic contact between physicians and pregnant women should be considered as a possibility to create a confidential and trustiness bond.

However, implementing a routine enquiry about DV during ANC it is not an easy task and requires several conditions and ethical principles that must be guarantee. Studies revealed that lack of time and knowledge about this topic were some barriers among health professionals to address DV. 21 Despite barriers and challenges evidences showed a positive attitude among women when questioned about DV experiences during ANC consultations. 22 For this reason, it is important implementing gender sensitive training among healthcare providers particularly obstetricians, gynecologists and residents, integrating DV education into medical school curricula and creating an institutional culture favorable to listen and offer an appropriate care to DV survivors. Technical preparation about how to ask and response is an essential knowledge, but DV is a sensitive topic that requires respectful of women's autonomy choices, non-judgmental, empathic, and confidential attitudes.

Additionally, it is observed an especial interest on examine effectiveness interventions to address DV during pregnancy. Thus, systematic reviews were conducted with this aimed showing a diversity of strategies including brief individualized consultation, referral to other professional and multiple therapy sessions. 5 23 Although the effectiveness of interventions still a literature gap due to lack of data and consistency in the outcomes 5 evidences suggest that not harmful effects were found due to interventions. 23 Furthermore, interventions for reducing and/or controlling DV among pregnant women conducted in low- and middle income countries were based in similar criteria such as women empowerment and contributing to identify resources and supporting women decisions to rise solutions according their particularly situation. 5 Hence, it is recommended that women centered-care response must to be the principal focus of interventions programs in healthcare settings.

Emergency and humanitarians' crises may present the risk of additional forms of violence and exacerbated conditions widely known as factors that increase women vulnerabilities. 15 24 Considering ANC as an essential service during Covid-19 pandemic context it is relevant to reinforce the importance of gynecologists and obstetricians to be aware to DV patterns and opportunities to offered first-line support to survivals.

Challenges such as strengthen and build capacities to implementing strategies to prevent DV in healthcare settings and especially during ANC, training healthcare providers and stakeholders, strengthen the representation of health sector as safety places for women and their children, change cultural norms which maintenance different forms of violence against women as an acceptable behavior are some critical points to strengthen and encouragement gynecologist and obstetricians to implementing routine screening about DV during pregnancy.
